# Rapid Facial Reactions in Response to Facial Expressions of Emotion Displayed by Real Versus Virtual Faces

**DOI:** 10.1177/2041669518786527

**Published:** 2018-07-12

**Authors:** Leonor Philip, Jean-Claude Martin, Céline Clavel

**Affiliations:** LIMSI, CNRS, University of Paris-Sud, Orsay, France

**Keywords:** rapid facial reaction, facial expressions, emotions, electromyography, virtual agent

## Abstract

Facial expressions of emotion provide relevant cues for understanding social interactions and the affective processes involved in emotion perception. Virtual human faces are useful for conducting controlled experiments. However, little is known regarding the possible differences between physiological responses elicited by virtual versus real human facial expressions. The aim of the current study was to determine if virtual and real emotional faces elicit the same rapid facial reactions for the perception of facial expressions of joy, anger, and sadness. Facial electromyography (corrugator supercilii, zygomaticus major, and depressor anguli) was recorded in 30 participants during the presentation of dynamic or static and virtual or real faces. For the perception of dynamic facial expressions of joy and anger, analyses of electromyography data revealed that rapid facial reactions were stronger when participants were presented with real faces compared with virtual faces. These results suggest that the processes underlying the perception of virtual versus real emotional faces might differ.

## Introduction

The realism of expressive faces and its impact on understanding emotions has been widely studied in the affective computing field of research (Garau et al., 2003; Nowak & Rauh, 2005). Expressive virtual faces are useful for conducting controlled experiments about the perception of facial expressions of emotions. Expressive virtual characters can be controlled precisely using multiple parameters such as gaze direction (Schrammel, Pannasch, Graupner, Mojzisch, & Velichkovsky, 2009; Soussignan et al., 2013) and posture (Mojzisch et al., 2006). Standardization of such parameters as the precise timing of dynamic facial expressions, the shape of the face, the distance between the eyes, facial symmetry and more generally the appearance of the presented faces decreases experimental biases. Some researchers have observed that these expressive virtual faces cause rapid facial reactions (RFRs; Joyal, Jacob, Cigna, Guay, & Renaud, 2014; Likowski, Mühlberger, Seibt, Pauli, & Weyers, 2008; Likowski, Mühlberger, Seibt, Pauli, & Weyers, 2011; Schrammel et al., 2009; Soussignan et al., 2013; Weyers, Mühlberger, Hefele, & Pauli, 2006; Weyers, Mühlberger, Kund, Hess, & Pauli, 2009). RFRs are considered as automatic facial responses and occur rather quickly when facial expressions are presented to participants (500 ms after the display of the facial expression; Dimberg, 1982; Dimberg, Thunberg, & Elmehed, 2000). RFRs enable to study low-level processes whereas a rating task enables to study only high-level processes.

Thus, the study of RFRs associated to emotional faces aims to shed new light on the theoretical debate about the understanding of social and emotional processing and the implications of attentional processes underlying emotion perception. Indeed, the appraisal theory suggests that RFRs might not be only motor responses but may also be the results of individual’s evaluation of important events or situations (Lazarus, 1991). Hess and Fischer (2013) questioned the matched motor hypothesis of emotional mimicry and preferred talking about “the Emotion Mimicry in Context Model.” According to these authors, RFRs depend on emotional intentions attributed to signals according to context. They hypothesize that spontaneous mimicry occurs only when a minimal form of affiliation or bonding exists between the observer and the target. This means that social interaction goals or inferred intentions should be neutral, and preferably affiliative, for emotional mimicry to occur. In a nonaffiliative context, RFRs would be incongruent with the emotional display.

Yet, few studies in social neurosciences consider the impact of the realism of expressive faces on the perception of emotions (Joyal et al., 2014; Moser et al., 2007; Mühlberger et al., 2009). Multiple studies used virtual faces to study human perception of emotional expressions. Virtual characters enable the design of controlled emotional stimuli to induce RFRs. However, it is not known whether the processes underlying the elicitation of RFRs are the same during the perception of virtual versus real human faces.

Thus, the study of RFRs associated to virtual faces aims to shed new light on (a) the theoretical debate about the understanding of social and emotional processing and the implications of attentional processes underlying emotion perception and (b) methodological implications about the use of virtual faces for studying how we perceive expressions of emotions.

In this study, we used virtual and real faces with static and dynamic expressions to compare RFRs produced by participants and examine the underlying processes. In addition, the nature of the processes involved in the perception of virtual versus real faces requires further investigations, particularly regarding how dynamic expressions are perceived compared with static expressions for both real and virtual faces.

### Animacy and the Perception of Facial Expressions

The perception of virtual or human faces involves cognitive, affective, and neural processes. The realism of faces can affect the social categorization of faces. For example, Balas (2013) observed that subjective ratings of animacy and gender categorization of real and artificial faces were impacted by the gender of the stimulus. Female faces are considered to look more artificial than male faces, and virtual faces seem to appear more feminine than real faces. Recent research highlights that people are able to make the difference between rapidly presented real and virtual faces when the identity of the individual is matched across test images (Balas & Tonsager, 2014). It has also been established that the eye region contributes to the determination of animacy and therefore helps distinguish virtual faces from real faces (Balas & Horski, 2012; Balas & Tonsager, 2014). Dyck et al. (2008) observed that facial expressions of fear and sadness were better recognized when expressed by virtual faces than when expressed by human faces. However, the facial expression of disgust was better recognized when displayed by human faces than when it was expressed by virtual faces. Joy and neutral expressions were also well recognized when expressed by humans and virtual characters, and the expression by a virtual character of joy and of a neutral expression did not cause a longer reaction time than the same expressions displayed by humans. Dyck et al.’s (2008) study suggests that the processes underlying the recognition of virtual versus real expressive faces might not require the same processing time.

In another study, Kätsyri and Sams (2008) explored the recognition of emotions expressed by virtual characters and real humans using dynamic expressions. Across all expressions displayed by virtual faces, dynamic expressions were better recognized when compared with static expressions. Yet, the authors did not observe this effect with dynamic expressions displayed by real humans. In the case of virtual faces, dynamic expressions of anger and disgust are better recognized than the corresponding static expressions. The authors did not observe this effect for the dynamic expressions of fear, joy, surprise, and sadness.

Experimental studies (Moser et al., 2007; Mühlberger et al., 2009) aiming at studying cerebral activity during the perception of emotional expressions conveyed by virtual faces or human faces showed that virtual faces are suitable for research about the processing of facial expressions. Yet, these studies also suggest that the processes involved in the perception of virtual faces might be different than the processes involved in the perception of human faces. According to Moser et al. (2007), virtual characters seem to be relevant stimuli for studying the perception of emotions because, like human faces, they cause similar amygdala activation. However, the activation of the fusiform gyrus only for human faces also suggests specific neural mechanisms for facial expressions of real humans. In addition, Mühlberger et al. (2009) suggest that the processes involved in the perception of virtual faces might require more attentional resources than the perception of faces of real humans. These studies suggest that the human visual system might be sensitive enough to distinguish real and virtual faces.

### Perception of Static and Dynamic Facial Expressions

Researchers have often used static emotional stimuli to investigate affective processes that occur during the perception of emotion (Dimberg, 1982; Ekman & Friesen, 1978). In real life, even though facial expressions of emotions are sometimes static (e.g., photos), they are most often dynamic. Recent data showed that dynamic facial expressions do not induce the same reactions for the observer as static facial expressions. Comparing how dynamic and static facial expressions of emotions are perceived enables us to better understand the underlying perceptual processes. In a behavioural neuroimaging study, Yoshikawa and Sato (2006) observed that dynamic facial expressions elicited stronger reports of emotional experience and a higher activation of the visual cortices, right inferior frontal gyrus and amygdala than static expressions. Recio, Sommer, and Schacht (2011) showed that dynamic facial expressions of happiness were better recognized compared with similar static facial expressions. These authors did not find any difference between static and dynamic facial expressions of anger. Torro Alves (2013) reported Fujimura and Suzuki’s (2010) Japanese study which used dynamic and static facial expressions of pleasant (joy, excitement, and relaxation) and unpleasant facial expressions (fear, anger, and sadness) presented in the central and peripheral visual fields. These authors observed that only dynamic expressions were better recognized in the peripheral region compared with static expressions. This suggests a greater sensitivity of this region to detect emotional salience linked to the movement.

A neuroimaging study showed specific activations in the right inferior occipital gyrus, right medial temporal gyrus, and right fusiform gyrus during the observation of dynamic facial expressions of happiness and fear (Sato, Kochiyama, Yoshikawa, Naito, & Matsumura, 2004). Moreover, Arsalidou, Morris, and Taylor (2011) observed that dynamic facial expressions induce more neural responses in regions related to emotional processing and appraisal of social cues compared with static expressions. Using dynamic and static expressions of anger and happiness with gaze directed toward the participant versus elsewhere, Sato, Kochiyama, Uono, and Yoshikawa (2010) found that the left amygdala was more activated when dynamic expressions were combined with the gaze directed toward the participant. This was not observed for static expressions.

### Static and Dynamic Facial Expressions: Electromyographic Studies

An electromyography (EMG) system enables researchers to measure spontaneous muscular facial activities during the perception of facial expressions of emotions. EMG studies indicate that looking at static facial expressions causes spontaneous RFRs that are congruent with the presented facial expressions (Dimberg, 1982).

Some studies have compared RFRs with EMG during the perception of dynamic versus static facial expressions. Sato, Fujimura, and Suzuki (2008) recorded the activity of the corrugator supercilii (associated with anger) and zygomaticus major (associated with happiness). They found that, in comparison to static expressions, dynamic facial expressions of anger enhanced the facial EMG activity of the corrugator, whereas dynamic expressions of happiness enhanced the facial EMG activity of the zygomaticus. Dynamic facial expressions caused more intense RFRs than static facial expressions. Similarly, Rymarczyk, Biele, Grabowska, and Majczynski (2011) observed that dynamic facial expressions of happiness and anger were rated as being more intense than the corresponding static facial expressions. These authors also observed that dynamic facial expressions of happiness caused higher activity of the zygomaticus major and lower activity of the corrugator supercilii than static facial expression of happiness. However, there was no significant difference in the activation of the corrugator supercilii when participants were presented with static versus dynamic expressions of anger. In another study involved the manual annotation of facial expressions using the facial action coding system (FACS; Ekman & Friesen, 1978): Sato and Yoshikawa (2007) discreetly filmed the faces of participants while they were watching dynamic and static expressions of anger and happiness. Afterwards, these authors coded the observed facial reactions using FACS, which enables to manually annotate the patterns of contraction or relaxation of the facial muscles. During the dynamic condition, facial muscles reacted in accordance with the observed expression, indicating that motions elicit similar spontaneous facial responses in observers. Sato and Yoshikawa (2007) showed that dynamic expressions not only elicit more pronounced facial mimic responses than static expressions, but these reactions can also be visually detected by an outside observer. Other studies about the perception of emotional facial expressions used virtual characters and studied their perception by observers (Weyers et al., 2006). These studies observed facial mimicry in response to virtual characters expressing emotional facial expressions. In addition, these characters have the same effect as dynamic faces of real humans and suggest that virtual dynamic characters enhance facial EMG activity compared with virtual static characters.

To our knowledge, there is, as yet, no comparative study about the impact of virtual versus real emotional faces on RFRs during the perception of facial expressions of emotions. The goal of the present study was to determine whether virtual and real emotional faces involve the same RFRs during the perception of facial expressions of emotions. In addition, we aim at a better understanding of the nature of the processes involved in the perception of virtual versus real faces, particularly regarding how dynamic expressions are perceived relative to static expressions for both real and virtual faces.

We suppose that our ability to distinguish real faces and virtual faces can affect RFRs because the literature suggests differences in the perception of facial expressions displayed by virtual characters versus real humans. As explained later, in our study, we used a model of three-dimensional facial appearance to create realistic computer-generated faces designed from the human faces depicted in photographs of real humans. Therefore, we have a matched set of real and virtual face stimuli. The present set of virtual face stimuli is thus composed of faces with variability in appearance that is approximately the same as the original human pictures.

## Methods and Materials

### Participants

Thirty-two French volunteers participated individually. They received a USB stick as compensation. Data from two participants had to be removed from the EMG analyses due to an exceptionally high numbers of trials with artefacts. Thus, EMG analyses were conducted with 30 participants (15 women) aged between 21 and 31 years (mean age = 23.1). Informed consent was obtained from all participants in written form after the experimental procedures had been explained.

### Experimental Design

The study had a 4 (Emotions: anger vs. happiness vs. sadness vs. neutral) × 2 (Realism: virtual human face vs. real human face) × 2 (Dynamism: static expression vs. dynamic expression) within-subjects design.

### Stimuli

The set of stimuli was composed of real or virtual human faces presented with pictures and video clips. Real human faces were selected from the Radboud database of validated expressive faces (Langner et al., 2010). We chose eight actors from this database (four women) expressing four emotions (joy, anger, sadness, and neutral). None of the faces was familiar to any of the participants. Each photo was framed in an oval shape.

Corresponding virtual faces were generated from these pictures with the FaceGen Modeller software (Krumhuber, Tamarit, Roesch, & Scherer, 2012; Roesch et al., 2011). This software, which has been used in several studies on the perception of facial expressions, enables the design of virtual faces that are quite close in appearance to the face of the original human. It is thus quite relevant for our study because it produces subtle differences in terms of realism between the virtual and the original human faces. The facial expressions were obtained by manipulating polygon groups on a three-dimensional mesh that made up the avatars’ facial structure. The polygon groups were comparable to the action units as described in the FACS (Ekman & Friesen, 1978). The following codes were used: 6 + 12 + 25 + 26 for happiness, 4 +5 + 24 for anger, and 1 + 4 + 15 for sadness. Neutral faces were used only static condition as control stimuli and no action unit was activated. The virtual faces were also framed in an oval shape.

Dynamic virtual and human facial expressions were generated with FantaMorph 5 software (Abrosoft FantaMorph, http://www.fantamorph.com; 33 fps) by linear interpolation. Each static and dynamic stimulus was presented for 1.5 seconds.

For the dynamic expression stimuli, 33 frames from neutral to emotional expressions were presented (i.e., one neutral image, 31 intermediate images, and the final emotional image). The frame rate was 22 images per second, and each clip last 1500 ms. The emotional expressions that corresponded to the final images (100%, i.e., the apex of the emotional expressions) in the dynamic condition were the static expression stimuli also presented during 1500 ms.

**Figure 1.**
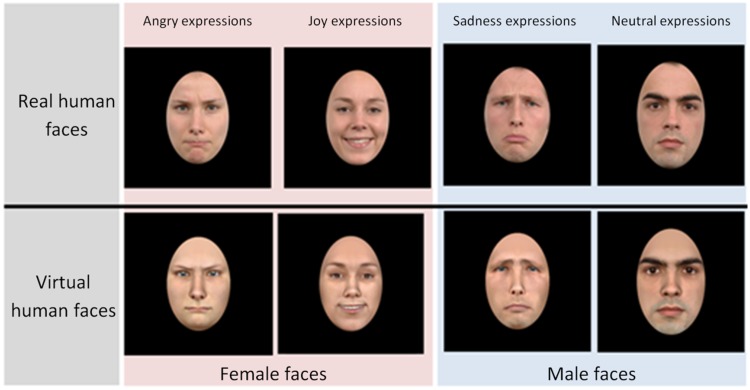
Examples of virtual and real human emotional facial expressions.

Each face (virtual human face or real human face) was shown with only one happy, neutral, sad, and angry expression, in static and dynamic conditions resulting in 112 expressions. Figure 1 shows examples of each emotion as expressed by a virtual human face or real human face.

### Procedure

After arriving at the laboratory and signing informed consent, participants sat at a distance of 0.6 meter of a LCD DELL UltraSharp 1907FP monitor. EMG electrodes were placed on the right side of the face of the participant (Dimberg et al., 2000). Corrugator supercilii, zygomaticus major, and depressor anguli activities were recorded. To avoid voluntary control of facial muscles (Fridlund & Cacioppo, 1986), participants were not informed of the purpose of the study nor of the recording of facial muscle activities. They were told that the study was about the analysis of facial temperature when viewing human emotional expressions.

**Figure 2.**
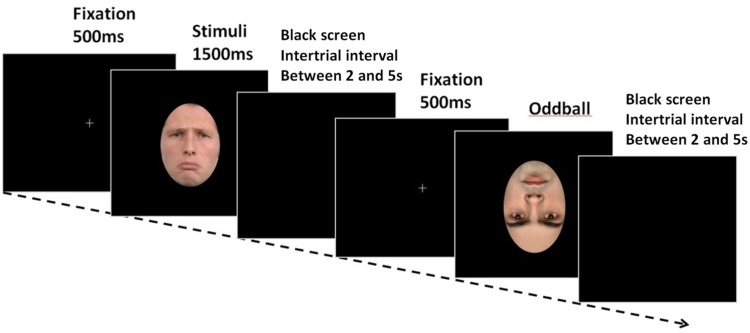
Example of a time course of a trial.

The participants were then introduced to the oddball task. The participants were instructed to key press only when the emotional face was displayed upside-down. It was an event that occurs infrequently and irregularly. Participants did not receive any feedback for correct or incorrect responses. Participants’ performance at the oddball task was not relevant to the predictions of the study; the task served as a means of ensuring that the participants were focusing on the target faces as they were presented on screen. All stimuli were displayed on the monitor using E-Prime 2.0 (Psychology Software Tools, PA). Each trial began with a central fixation cross (1000 ms), followed by a facial expression displayed by a real human face or a virtual human face during 1500 ms. A black screen was displayed during 2- to 5-s intertrial intervals. The order of stimuli presentation was randomized across participants. Figure 2 shows an example of a time course of a trial.

Debriefing after the experience showed that no participant had suspected that facial muscle reactions were indeed measured.

### Apparatus

The stimuli were presented on a 19-in. LCD DELL monitor (UltraSharp 1907FP; color TFT active matrix LCD; 1280 vertical × 1024 horizontal pixels resolution, 60 Hz refresh rate) from a viewing distance of about 0.6 m. The stimuli were presented at 609 pixels in height × 658 pixels in width.

### EMG Recording

Using Brain Products acquisition system, EMG activity was continuously recorded using SensorMedics 4-mm shielded Ag/AgCl miniature electrodes. The target areas of the right side of the face were cleaned with alcohol and gently rubbed to reduce interelectrode impedance. Two pairs of electrodes filled with electrolyte gel were placed on the target area and secured using adhesive collars and sticky tape. Following the guidelines proposed by Fridlund and Cacioppo (1986), the two electrodes in each pair were placed approximately 1.5 cm apart over two muscle regions associated with different emotional expressions. For example, the activity of the left corrugator supercilii muscle, which lowers brows, is associated with anger. This corrugator is involved in several negative facial expressions (Dimberg, 1988). Activity of the depressor anguli, which move the corners of the mouth downward, is implicated in sadness and was also recorded. Activity over the left zygomaticus major muscle, which pulls lip corners up, is usually associated with pleasure or happiness and was recorded. The ground electrode was placed on the upper right forehead. The signal was band-pass filtered online between 10 and 500 Hz and a 50-Hz notch filter, rectified, and smoothed online using a 500 ms time constant. EMG trials containing artefacts were manually rejected. No more than 15% of the trials were removed per muscle. Integral values were subsampled offline at 10 Hz and log transformed to reduce the impact of extreme values. To allow for comparisons, values were then standardized over the number of participants and over the number of muscles. Temporal profiles of facial EMG during the first 1000 ms following stimulus onset were investigated by calculating the mean amplitudes during 10 time intervals of 100 ms. The prestimulus values (computed over 200 ms before the stimuli onset) were then subtracted from the poststimulus activity to measure the activity caused by the perception of each stimulus (i.e., to calculate the change from baseline). EMG activity was thus defined as the change from baseline occurring between 0 and 1000 ms after the stimulus onset. Finally, the mean levels of corrugator, zygomaticus, and depressor activity were computed separately. We averaged two time windows (100–500 ms and 500–1000 ms; Moody, McIntosh, Mann, & Weisser, 2007) because RFRs appear after 500 ms after the stimulus (Dimberg, 1982; Dimberg, Hansson, & Thunberg, 1998).

### Statistical Analysis

The normality of the collected data was verified by the Shapiro–Wilk test. Experimental effects on the average EMG activity were tested with repeated-measures analysis of variance (ANOVA) with factors Emotion (4 levels) and Realism (2 levels) and Dynamism (2 levels). ANOVAs were calculated using SPSS 17.0. All post hoc comparisons were Bonferroni corrected. The results presented in this section were considered statistically signiﬁcant when *p* < .05.

## Results

### Impact of Dynamism, Realism, and Emotions on Corrugator Activity

A three-way Dynamism × Emotion × Realism ANOVA on the corrugator data over a window of 500 to 1000 ms revealed a significant effect of Emotion, *F*(29, 3) = 3.24, *p* = .04; $\eta_{\text{p}}^{2}$ = .14, a Dynamism × Emotion interaction, *F*(29, 7) = 2.75, *p* = .02; $\eta_{\text{p}}^{2}$ = .19.

#### Impact of emotions on corrugator activity for dynamic and real expressions

The ANOVA on the corrugators data over a window of 500 ms to 1000 ms revealed a significant main effect of Emotion, *F*(29, 2) = 4.59, *p* = .02; $\eta_{\text{p}}^{2}$ = .14. Post hoc analyses revealed that the corrugator was more activated during the perception of angry expressions than during the perception of joy expressions (*p* = .02).

#### Impact of emotions on corrugator activity for static and real expressions

A significant effect of Emotion, *F*(29, 3) = 2.95, *p* = .05, was observed: The corrugator was more activated for angry expressions than for joy (*p* = .03) and neutral expressions (*p* = .01).

#### Impact of emotions on corrugator activity for dynamic and virtual expressions

An Emotion effect (*p* = .03) revealed that the corrugator was more activated for the angry expressions than for the joy (*p* = .001) and neutral expressions (*p* = .03).

#### Impact of emotions on corrugator activity for static and virtual expressions

**Figure 3.**
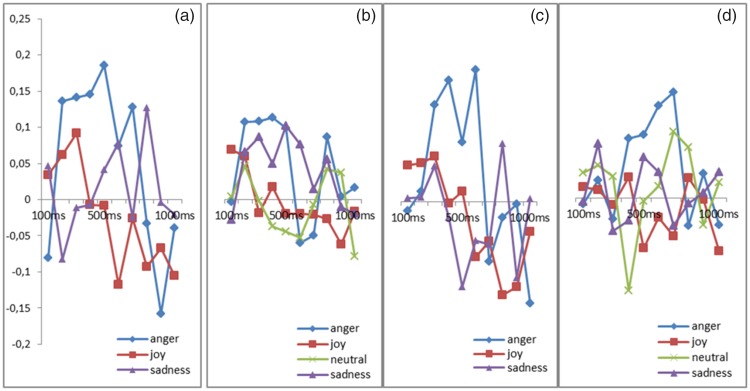
(a) Activity of corrugator supercilii during the perception of real human dynamic expressions of anger, joy, and sadness. (b) Activity of corrugator supercilii during the perception of real human static expressions of anger, joy, sadness, and neutral expression. (c) Activity of corrugator supercilii during the perception of virtual human dynamic expressions of anger, joy, and sadness. (d) Activity of corrugator supercilii during the perception of virtual human static expressions of anger, joy, neutral, and sadness.

A trend Emotion effect (*p* = .05) revealed that the corrugator was more activated for the angry expressions than for the joy expressions (*p* = .02; Figure 3).

#### Impact of dynamic or static and real or virtual anger expressions on corrugator activity

**Figure 4.**
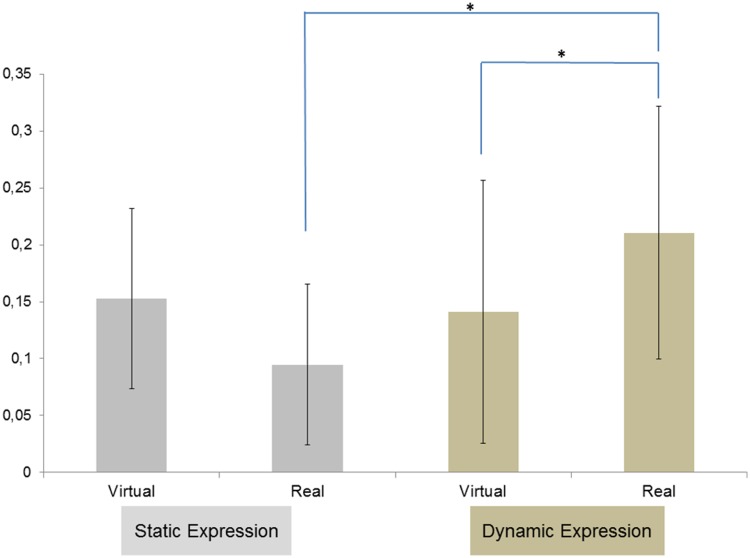
Mean EMG change activity for M. corrugator supercilii in response to real versus virtual faces, static versus dynamic anger facial expressions. Asterisks indicate the signiﬁcant effects, indicating higher activity for real faces than for virtual faces in dynamic condition and higher activity for dynamic expression than for static expression in real face condition.

Post hoc tests on corrugator data showed that dynamic anger expressions caused greater activities of the corrugator than static anger expressions, *t*(29) = 4.87, *p* < .001. In addition, the corrugator was also more activated for real dynamic anger expressions than for virtual dynamic anger expressions, *t*(29) = 3.21, *p* = .01. The corrugator was also more activated for the real dynamic anger expressions than for real static anger expressions, *t*(29) = 3.21, *p* = .01 (Figure 4).

### Impact of Dynamism, Realism, and Emotions on Zygomaticus Activity

Three-way Dynamism × Emotion × Realism ANOVA on the zygomaticus data over a window of 500 to 1000 ms revealed a significant main effect of Emotion, *F*(29, 3) = 4.56, *p* = .01; $\eta_{\text{p}}^{2}$ = .20, a Dynamism × Emotion interaction, *F*(29, 7) = 2.21, *p* = .06; $\eta_{\text{p}}^{2}$ = .18, and an Emotion × Dynamism × Realism interaction, *F*(29, 15) = 2.45, *p* = .02; $\eta_{\text{p}}^{2}$ = .22.

#### Impact of emotions on zygomaticus activity for dynamic and real expressions

The ANOVA on the zygomaticus data over a window of 500 ms to 1000 ms revealed a significant main effect of Emotion, *F*(29, 2) = 5.60, *p* = .02; $\eta_{\text{p}}^{2}$ = .20. The zygomaticus was more activated during the perception of joy expressions than during the perception of angry expressions (*p* = .02) and sadness expressions (*p* = .04).

#### Impact of emotions on zygomaticus activity for static and real expressions

A significant effect of Emotion (*p* = .03) was observed: The zygomaticus was more activated for joy expressions than for angry (*p* = .02), sadness (*p* = .01), and neutral expressions (*p* = .02).

#### Impact of emotions on zygomaticus activity for dynamic and virtual expressions

An Emotion effect, *F*(29, 2) = 3.29, *p* = .05; $\eta_{\text{p}}^{2}$ = .14, revealed that the zygomaticus was more activated for joy expressions than for angry expressions (*p* = .05).

#### Impact of emotions on zygomaticus activity for static and virtual expressions

**Figure 5.**
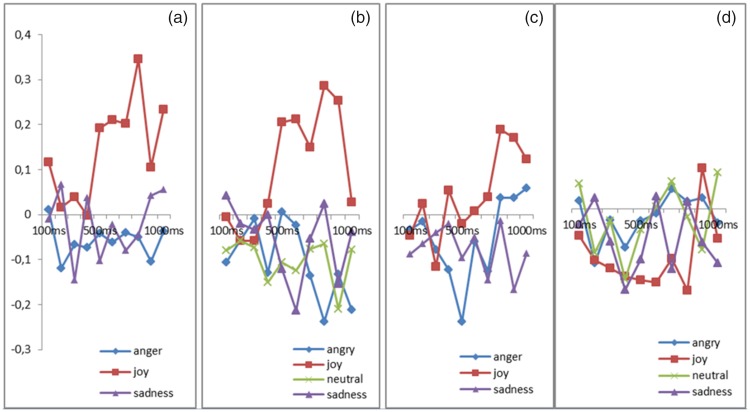
(a) Activity of zygomaticus major during the perception of real human dynamic expressions of anger, joy, and sadness. (b) Activity of zygomaticus major during the perception of real human static expressions of anger, joy, sadness, and neutral expression. (c) Activity of zygomaticus major during the perception of virtual human dynamic expressions of anger, joy, and sadness. (d) Activity of zygomaticus major during the perception of virtual human static expressions of anger, joy, neutral, and sadness.

There was no Emotion effect on the zygomaticus activity (Figure 5).

#### Impact of dynamic or static and real or virtual joy expressions on zygomaticus activity

**Figure 6.**
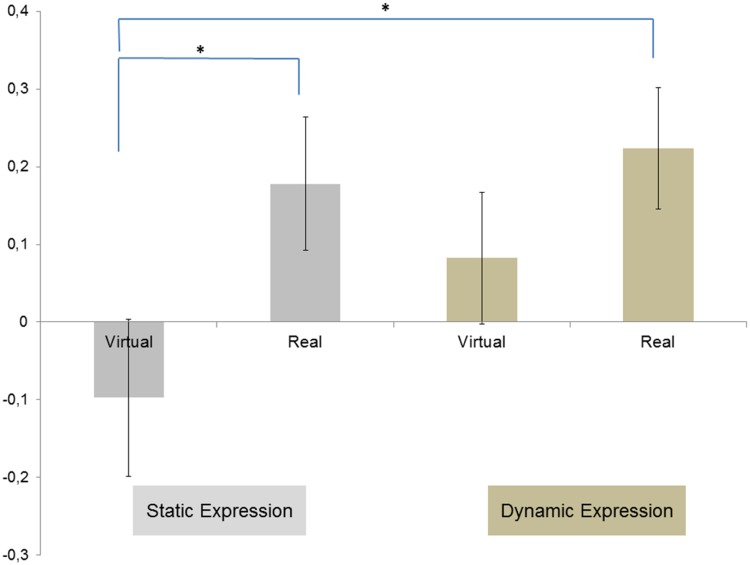
Mean EMG change activity for M. zygomaticus major in response to real versus virtual faces, static versus dynamic facial expressions of joy. Asterisks indicate the signiﬁcant effects, indicating higher activity for real face than for virtual face in static condition and higher activity for real faces in dynamic condition than for virtual faces in static condition.

Post hoc tests on zygomaticus data showed that dynamic facial expressions of joy caused a greater activity of the zygomaticus than static expressions of joy, *t*(29) = 3.25, *p* = .01. In addition, the zygomaticus was also more activated for real dynamic expressions of joy than virtual static expressions of joy, *t*(29) = 4.19, *p* < .001. The zygomaticus was also more activated for real static expressions of joy than virtual static expressions of joy, *t*(29) = 3.61, *p* < .001 (Figure 6).

### Impact of Dynamism, Realism, and Emotions on Depressor Activity

A three-way Dynamism × Emotion × realism ANOVA using EMG data, on a window from 500 to 1000 ms, revealed a trend effect of Emotion, *F*(29, 3) = 2.64, *p* = .07; $\eta_{\text{p}}^{2}$ = .25, but showed no interaction effect between the different factors.

#### Impact of emotions on depressor activity for dynamic and real expressions

The ANOVA on the depressor data over a window of 500 ms to 1000 ms revealed a significant main effect of Emotion, *F*(29, 2) = 3.60, *p* = .02; $\eta_{\text{p}}^{2}$ = .22. The depressor was more activated during the perception of sadness expressions than during the perception of joy expressions (*p* = .01).

#### Impact of emotions on depressor activity for static and real expressions

A significant effect of Emotion (*p* = .04) revealed that the depressor was more activated for sadness expressions than for expressions of joy (*p* = .05).

#### Impact of emotions on depressor activity for dynamic and virtual expressions

An Emotion effect (*p* = .05) revealed that the depressor was more activated for expressions of sadness than for expressions of anger (*p* = .03) and expressions of joy (*p* = .01).

#### Impact of emotions on depressor activity for static and virtual expressions

**Figure 7.**
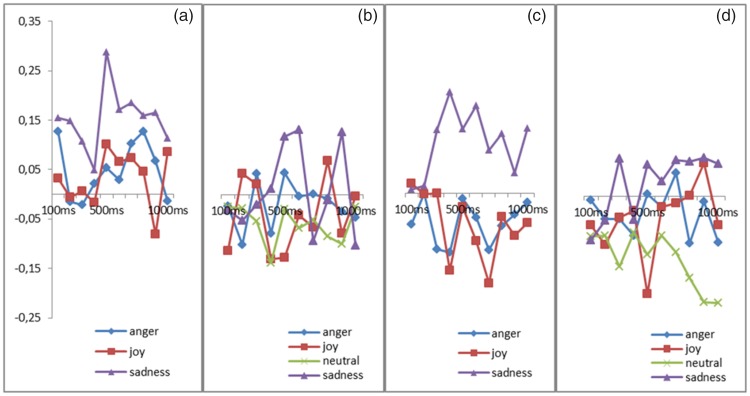
(a) Activity of depressor anguli during the perception of real human dynamic expressions of anger, joy, and sadness. (b) Activity of depressor anguli during the perception of real human static expressions of anger, joy, sadness, and neutral expressions. (c) Activity of depressor anguli during the perception of virtual human dynamic expressions of anger, joy, and sadness. (d) Activity of depressor anguli during the perception of virtual human static expressions of anger, joy, neutral, and sadness.

There was no Emotion effect on the activity of the depressor (Figure 7).

## Discussion

### Facial Mimicry Based on Human or Virtual Faces

As expected, facial EMG data revealed congruent facial muscular reactions to the emotional expressions displayed by a real or virtual human. These results support the facial mimicry hypothesis and are in line with previous results (e.g., Dimberg, Thunberg, & Grunedal, 2002). Whatever the real or virtual nature of stimuli, viewing happy faces induced an activation of the zygomaticus major muscle, whereas viewing angry faces produced increased corrugator supercilii activities and finally viewing sad faces produced increased depressor.

### Muscle Activity Elicited by Dynamical Expressions

The observed activity of the corrugator and zygomaticus muscles suggests that dynamic facial expressions of anger and joy cause more intense congruent RFRs than static facial expressions of the same emotions. We observed the same pattern for virtual and real human faces: There is thus a dynamism effect for both virtual and real faces. Dynamic facial expressions, whether real or virtual, raised greater RFRs than the corresponding static facial expressions. This is consistent with studies that show a dynamism effect for human expressive faces (Rymarczyk, et al., 2011; Sato et al., 2008) and for virtual expressive faces (Weyers et al., 2006, 2009). More specifically, for the perception of anger expressions, the results suggest that real dynamic expressions cause greater RFRs than virtual dynamic expressions. The perception of joy expressions causes greater RFRs for static real expressions than static virtual expressions. In addition, the perception of a dynamic real expression of joy causes greater RFRs than a static virtual expression of joy.

### Muscle Activity According to Virtual or Real Human Faces

The RFRs caused by the perception of angry expressions are also affected by the nature of the stimulus. Specifically, for the perception of dynamic expressions of anger, the RFRs were greater in response to real human faces than virtual human faces. For the joy expression, the RFRs were also affected by the nature of the stimulus but only for static expressions. The perception of static expressions of joy caused greater RFRs in response to real human faces than virtual human faces.

The facial EMG data revealed three important issues. First, we found that participants displayed congruent facial reaction to others’ facial emotional expressions (mimicry) whatever the nature of stimuli (real or virtual) and the dynamism of stimuli (static or dynamic). Second, the dynamism of stimuli influences the RFRs. When the stimulus is dynamic, participants tend to react more intensely than when the stimulus is static. And third, the dynamism effect is even stronger when the emotional human expression is real.

These results provide insights about the relevance of virtual characters for the study of affective processes in emotion perception and encourage discussing the implications of using virtual faces rather than real faces to study human cognitive processes such as emotion perception.

Static faces are sometimes considered an impoverished representation of real stimuli because facial expressions are intrinsically dynamic (Fiorentini & Viviani, 2011). In this perspective, we consider that dynamic facial expressions are more similar to natural stimuli, and therefore induce a greater ecological validity (Ambadar, Schooler, & Cohn, 2005). In the same way, we can suppose that virtual faces are also an impoverished representation of real stimuli and do not provide an ecological validity. Mühlberger et al. (2009) recall that virtual face are often created based on prototypical faces of a male or female and differ according in hair types and hair colours. Thus, the structural features of these faces remain notably stable over the entire experimental setup. Unlike real human face, these virtual faces are symmetrical. Finally, Mühlberger et al. (2009) suggest that virtual expressive faces provide less “perceptual noise” than real expressive faces.

To our knowledge, this study is the first to compare real and virtual human expressions based on the same graphic model of the same person. Previous studies involved either real human faces or virtual human faces, but never both. Studies which used virtual human faces hypothesized that the mechanisms underlying the perception of emotion are the same when we perceive virtual human expressions than when we perceive real human expressions.

Virtual characters enable generating controlled emotional stimuli to induce RFRs. They seem to feature the same dynamism effect as real human expressive faces. However, in keeping with functional magnetic resonance imaging and electroencephalographic data (Moser et al., 2007; Mühlberger et al., 2009), our results show the level of facial mimicry varies according to the nature (real vs. virtual) of stimulus. The facial mimicry is higher when the stimulus is real than when the stimulus is virtual. Thus, a better understanding of the amplitude difference in muscular activity generated by virtual or real faces could allow a better understanding of the mechanisms underlying the perception of emotions.

One problem in studies comparing virtual and human faces is the inconsistency of the degree of realism of virtual faces across studies. Researchers do not use the same software to design virtual characters. They also do not manipulate or produce the same level of realism. Yet, the level of realism of these facial expressions may influence the perception of emotions, and thus the resulting RFRs produced by participants. One possible future direction would be to design and use additional virtual faces with a different realism levels and study the RFRs they elicit. The goal would be to examine whether the level of realism of the character and expressiveness influence the intensity of RFRs.

Finally, recent studies draw attention to the importance of social contexts in emotional perception and especially for facial mimicry (Bourgeois & Hess, 2008). Soussignan et al. (2013) showed that when the participants viewed the stimuli passively, their facial muscle responses may rapidly match to the senders’ facial displays according to their appraisal of their relation to others. Hess and Fischer (2013) questioned the *Matched Motor Hypothesis* of emotional mimicry and preferred talking about “the Emotion Mimicry in Context Model.” According to these authors, RFRs depend on emotional intentions attributed to signals according to the context. Van der Schalk, Hawk, Fischer, and Doosje (2011) showed that emotional mimicry varies according to the group membership of stimulus. Thus, the facial mimicry is higher when the stimulus is displayed by ingroup members than when the stimulus is displayed by outgroup members. Another problem in emotional perception studies using virtual faces is how these stimuli are interpreted. One possible explanation would be that social expectations for virtual faces and real faces would be different. Virtual faces could be regarded as outgroup members. A future study could investigate these questions as well as if participants perceived the difference between virtual and real faces.

In addition, Guerra, Sánchez-Adam, Anllo-Vento, and Vila (2012) showed that a zygomatic major response varies according to the familiarity level of the stimulus. The zygomatic major activity is higher when the stimulus is displayed by loved familiar faces (partner, father, mother, and best-friend) than when the stimulus is displayed on unknown faces. Future experiments might reveal whether similar results would be obtained when the virtual face represents a familiar face versus an unknown face. Such comparison could be helpful to manipulate the degree of closeness between the participant and the virtual agent.
